# Building a Low-Cost Wireless Biofeedback Solution: Applying Design Science Research Methodology

**DOI:** 10.3390/s23062920

**Published:** 2023-03-08

**Authors:** Chih-Feng Cheng, Chiuhsiang Joe Lin

**Affiliations:** Department of Industrial Management, National Taiwan University of Science and Technology, Taipei 106335, Taiwan

**Keywords:** human–robot interaction, low-cost, biometric, affective computing, wearable sensor, user experience

## Abstract

In recent years, affective computing has emerged as a promising approach to studying user experience, replacing subjective methods that rely on participants’ self-evaluation. Affective computing uses biometrics to recognize people’s emotional states as they interact with a product. However, the cost of medical-grade biofeedback systems is prohibitive for researchers with limited budgets. An alternative solution is to use consumer-grade devices, which are more affordable. However, these devices require proprietary software to collect data, complicating data processing, synchronization, and integration. Additionally, researchers need multiple computers to control the biofeedback system, increasing equipment costs and complexity. To address these challenges, we developed a low-cost biofeedback platform using inexpensive hardware and open-source libraries. Our software can serve as a system development kit for future studies. We conducted a simple experiment with one participant to validate the platform’s effectiveness, using one baseline and two tasks that elicited distinct responses. Our low-cost biofeedback platform provides a reference architecture for researchers with limited budgets who wish to incorporate biometrics into their studies. This platform can be used to develop affective computing models in various domains, including ergonomics, human factors engineering, user experience, human behavioral studies, and human–robot interaction.

## 1. Introduction

Biofeedback involves using electrical instruments to measure a person’s biometric responses, including brainwaves, heart rate, skin conductance, facial expressions, respiration, peripheral skin temperature, and muscle tone [1,2]. These biometric signals are often referred to as physiological [3,4,5] and psychophysiological signals [6]. The applications of biofeedback are broad and include medical purposes, such as physical and occupational therapy [7,8,9,10], psychological clinics [11,12,13,14], and cognitive research [15,16,17,18]. To ensure the accuracy and quality of their work, researchers often use medical-grade devices in biofeedback studies. The results obtained from medical-grade equipment provide a comparable standard for other research, which is particularly important for medical applications. Therefore, the use of medically certified instruments in therapeutic studies is mandatory. However, the high price of such equipment can be a significant barrier to its widespread adoption. Manufacturers must acquire an FDA class II or CE IIa certification for their products to meet medical certification requirements, making them excellent and reliable, but costly.

### 1.1. Consumer-Grade Physiological Sensors Applied in Research

Cost can be a concern when using medically certified devices for non-medical purposes [19]. In recent years, consumer-grade biometric devices have been extended to non-medical fields, such as measuring pupils’ attention in education [20], brain–computer interfaces in human–computer interaction [21], workers’ mental load in human–robot collaboration [22], and affective computing [23,24]. Some consumer-grade biometric devices have been reported to be as accurate as medical-grade products [25,26] or can be used for health care purposes [27].

When selecting wearable sensors for Industry 5.0 applications, the two top features to consider are system development kit (SDK) support for real-time data streaming and wireless communication protocols. Three communication protocols are widely used in the industry sector. Experts recognize that the Bluetooth Low Energy (BLE) protocol is much more important than ANT+ and Wi-Fi. Every wearable device on the market supports BLE, the mainstream communication protocol. Other features, such as weight and comfort for workers without obstructions, are also important considerations [28].

Photoplethysmography (PPG) is prevalent in the application of individual health care [27]. Bolanos et al. [29] indicated that heart rate variability (HRV) derived from PPG has excellent potential to replace the one from ECG. Recently, no significant differences have been reported in the HRV features derived from PPG and ECG signals in time, frequency, and non-linear domains [30]. However, PPG should be used only when the user is resting [31,32] due to motion artifacts caused by the movement of the PPG sensor over the tissue, skin deformation due to muscle contraction, and blood flow dynamics [33,34]. Nevertheless, an algorithm can reduce movement artifacts using user kinematics data from embedded accelerometers [35]. PPG is a non-invasive, low-cost, and wearable wireless device that can be an alternative to electrocardiogram (ECG) technology for heart rate (HR) monitoring. Although ECG has been continuously improved in terms of measurement accuracy and wearing comfort, the flexibility, portability, and convenience for users have not been enhanced [27]. In contrast, PPG does not require several electrodes to be placed on specific body locations. Users can wear a PPG device like a watch, with flexibility of movement, portability to any location, and convenience to monitor HR all day. These features make PPG suitable for non-medical activities [27], such as user experience studies [36]. In recent years, there has been an extension of the use of PPG to non-medical fields, such as measuring pupils’ attention in education [20], brain–computer interfaces in human–computer interaction [21,22], and affective computing [23,24]. Some consumer-grade biometric devices have been reported to be as accurate as medical-grade products [25,26] and can be used for health care purposes [27].

Two review papers [19,36] provide a list of consumer-grade EEG devices and a comprehensive literature survey for researchers’ reference. The key results are summarized below: four brands, namely, NeuroSky, Emotive, Interaxon, and OpenBCI, provide consumer-grade EEG products with high potential. Most EEG products are equipped with dry sensors, single channels, and BLE or classical Bluetooth communication protocols to stream data. The sampling frequency ranges from 128 to 512 Hz. The classification accuracy of the machine learning algorithm for the responded study ranges between 60% to 90%, as cited in the literature in the categories of cognition, education, entertainment, and brain–computer interaction. When comparing the performance of the power spectra, NeuroSky MindWave is similar to two medical-level EEG devices. Emotive EPOC is worse than the benchmark medical-level rival, Interaxon Muse, which demonstrates lower reliability than medical-level products. The results for OpenBCI resemble those of medical-grade devices. Moreover, a random time lag is a common phenomenon in wireless EEG devices.

Consumer-grade electroencephalography (EEG) products have been widely accepted for non-medical applications [19,36]. Similarly, PPG devices are popular for non-medical health care [37,38]. Innovations in technology over the last decade have advanced consumer-grade wearable sensors, enabling them to prosper in the market. The wireless, wearable, and lightweight features make consumer-grade products capable of collecting data continuously with no time or location limits. As a result, users do not feel discomfort after wearing such devices for a long period. In addition, they are easy to use and even novices can handle them easily.

In contrast, a medical-grade EEG system is accurate, but it comes at the price of a complex structure and long setup time [36]. ECG does not offer users the flexibility, portability, or convenience offered by medical-grade EEG [27]. Consequently, medical-grade device applications are usually limited to the laboratory [19,36].

Another point to note is that for research requiring high data retention, reliable communication, no real-time data transmission, and involving field investigations, wearable devices with temporary memory storage should be considered, with data uploaded to cloud servers at regular intervals. Examples of such research include investigations of the relationship between human well-being and daily experiences [39] or the relationship between human emotions and daily life events [40]. Physiological signals are excellent objective metrics for such research, and when combined with subjective assessments from participants, they can provide valuable insights. However, such studies involve scenarios that are not pre-designed or controlled; therefore, experiments can only be conducted in real-life settings. Carrying around another real-time data collection system is not feasible.

### 1.2. Relationship between User Experience and Users’ Emotional State

Norman coined and proposed the term “user experience” [41]; however, initially, this term lacked a clear definition. Years later, Norman and Nielsen defined UX as “meeting the exact needs of the customer” and “a joy to own, a joy to use” [42]. Some experts organized a special interest group (SIG) to comprehensively investigate UX. There are many definitions of UX in the survey results of the published literature or on the websites of UX organizations [43]. The conclusion of the SIG suggests that researchers may choose their preferred definition from the identified list, which includes Norman and Nielsen’s definition.

The evaluation of UX traditionally uses participants’ self-evaluation through a questionnaire or personal interview after experiencing a product. This assessment method relies on the participants’ subjective perceptions, and is referred to as subjective evaluation. In contrast, UX evaluation based on biometrics is considered an objective method as it uses signals generated from the human autonomic nervous system. The SIG mentioned in the previous paragraph identified eighty-six UX evaluation tools [44], but only four of these use physiological signals or facial expressions to assess UX. Therefore, thirty-three subjective methods utilize emotion/affect/hedonic as the index for UX evaluation. This finding is consistent with Norman and Nielsen’s definition, and implies that UX is related to a user’s emotional state after experiencing a product. Moreover, the limited number of tools using objective methods suggests that researchers should investigate this topic in greater depth.

### 1.3. Affective Computing as a Tool for User Experience Evaluation

Affective computing [45] is an algorithm that recognizes a human’s emotional state [46] through biometric signals [47]. Accordingly, a user’s perceived pleasure while experiencing a product estimated using biometrics might serve as a UX metric. When a user experiences pleasurable emotions such as joy or positive valence for a product, it is reasonable to believe that this product generates a good user experience. Conversely, a user responding with anger or negative valence signals a poor user experience if. Instead of relying on a user self-report assessment or verbal expressions––referred to as subjective evaluation, and used in traditional UX studies––the affective computing applied in UX research is considered an objective method [46]. Consequently, researchers interested in user experience (UX) use affective computing to recognize users’ emotional states when they experience a specific software or product [48,49,50,51,52].

Although subjective methods are related to the assessment target, the participants may have cognitive bias [53] and may not be sufficiently robust [54]. An objective assessment could compensate for this disadvantage [55]. Thus, subjective measures should not be the sole metrics used to evaluate UX [49]. Using a subjective assessment of human emotion may be unreliable since emotions are often swift, hard to perceive, and sometimes have multiple states [56]. Additionally, participants may be afraid to confess their emotions to the researcher. Worse, some participants may answer questions by imagining what the researcher expects them to say [56].

In contrast, an objective metric could assist researchers to fill the gap caused by using subjective methods to evaluate the UX of a specific product. For example, a case study evaluated participants’ user experience of three different virtual dressing websites using verbal expressions and biometrics [56]. There was no difference between the websites in terms of positive expressions resulting from verbal expressions. However, the percentage of engagement and attention, the positive/negative emotion, and the joy derived from biometrics showed that the three websites differed. This result illustrates the value of biometrics.

Moreover, affective computing can be applied in robotics to increase people’s enjoyment while interacting with robots. Physiological signals were one of the elements acting as medical robots’ human–machine interface. Using flexible electronics and devices makes the interface biocompatible, functional, conformable, and low-cost, resulting in an excellent user experience [57]. Service robots in the health care sector can aid patients with cognitive obstruction via built-in affective computing algorithms [58]. In commerce, empowering service robots with emotion recognition is a highly popular topic in research [59,60,61,62,63,64]. Some emotion recognition databases are available for service robots [65,66]. Accordingly, users’ emotional state during the experience with robots is a critical metric of human–robot interaction [67,68,69]. Affective computing algorithms built into robots allow machines to recognize humans’ emotional states.

The present study focused on single-electrode electroencephalography (EEG), photoplethysmography (PPG) technology in heart rate (HR), galvanic skin response (GSR), and facial expressions, which are the biometrics frequently applied in affective computing studies [4,70,71,72,73,74,75,76,77,78,79,80,81,82,83,84].

Although subjective methods are commonly used for UX assessment, they have some limitations. For example, participants may have cognitive biases [53] or the methods may not be sufficiently robust [54]. To compensate for these limitations, objective assessments could be used [55]. Therefore, subjective measures should not be the only metric used for UX evaluation [49]. Assessing human emotions subjectively can also be unreliable since emotions can be swift, hard to perceive, and have multiple states [56]. Additionally, participants may be reluctant to reveal their emotions to the researcher, and some participants may answer questions based on what they think the researcher expects them to say [56].

### 1.4. Motivations and Objectives

This research aims to assist researchers who are interested in applying affective computing to investigate human behavior but are limited by a restricted budget for medical-grade instruments. While various consumer-grade alternatives are available, there are drawbacks to using these commercial products. Firstly, most consumer devices only provide a single biometric signal, which means that a study typically requires multiple physiological signals from different manufacturers. This means the software is not an integrated system, such as that used in medical-grade devices. Therefore, researchers must use multiple, specific software programs simultaneously to collect data. Consequently, each instrument may need an independent computer or mobile device, and software manipulation is more complex than with an integrated system. Moreover, it is impossible to synchronize data from different consumer alternatives in terms of timestamps, even with multiple experimenters collaborating to press the buttons simultaneously. This can lead to a minimal time gap of less than one second, which may be insufficient for some psychology studies. Finally, the data processing of different signals distributed in many files can also be troublesome.

The objective of this research is to use affordable hardware and free software libraries to develop a low-cost biofeedback platform for biometrics-related studies, such as human factors engineering, user experience, human behavioral studies, and human–robot interaction. The specific aim of the hardware is achieved by using consumer products or electronic modules designed for Arduino makers rather than medical-grade instruments. Another objective of the software is achieved through the use of open-source libraries that can be used free of charge. The software is an object-oriented programming (OOP) class that serves as a software development kit (SDK), which can be reused for other research or as a standalone biometrics collecting system. An SDK design allows researchers to integrate data collection with their experimental stimulus into a single system through customized coding. Nonintegrated devices require at least one additional computer beside the one controlling the experiment stimulus [85]. Moreover, the integrated software simplifies researchers’ manipulation during experimentation. A single button click triggers the stimulus, and the data collection is synchronized. Therefore, the biofeedback platform developed in this study improves the ease of operation in experiments and reduces equipment costs.

## 2. Methods

Design science research (DSR) is a problem-solving paradigm achieved through the invention of innovative artifacts [86]. DSR has been adopted as a legitimate research paradigm in the information system research community to develop innovative software [87]. The design science research methodology (DSRM) is the most commonly applied model in research communities [86]. One possible entry point in DSRM is problem-centered initiation, where the ultimate goal is to design and develop an artifact for the identified problem. This study aims to solve a problem that many junior researchers encounter by developing a reusable hardware/software architecture. Accordingly, the DSRM framework is a suitable paradigm for this study.

**Figure 1:**
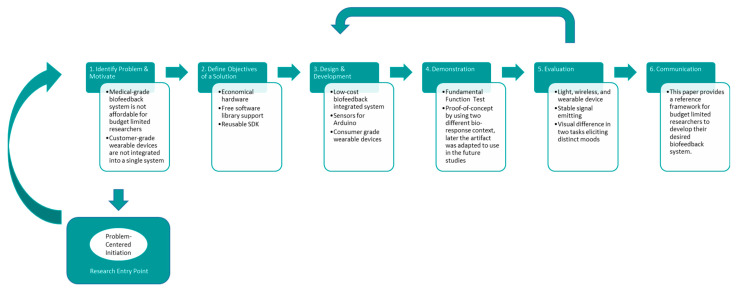
The development procedure of the low-cost biofeedback platform using the design science research methodology.

Figure 1 shows the development procedure of the low-cost biofeedback system in this study. The DSRM paradigm involves six activities, with activity one being to identify the problem and motivate, as stated in Section 1.4. Similarly, the objectives of this paper, defined in activity two are also described in Section 1.4. The design and development, demonstration, and evaluation in activities three to five are presented in the results section below. The last activity, communication, is the purpose of this paper.

## 3. Results

### 3.1. Using Biofeedback Sensors with Arduino

#### 3.1.1. Activity 3: Design and Development

During the first stage of hardware selection, biofeedback sensors for Arduino were identified as the preferred choice due to their cost-effectiveness. Table 1 presents the survey results of the available sensors in Taiwan’s makers market. These sensors can be purchased from local suppliers or manufacturers’ websites worldwide.

Many third-party companies manufacture EEG solutions using only NeuroSky’s ThinkGear ASIC Module (TGAM). The TGAM module generates EEG signals automatically, while its EEG module board integrates a classical Bluetooth module, HC-06, which makes it easy to connect to a personal computer. This allows users to easily access the EEG signals via the PC’s COM port. The TGAM module provides eight spectrum signals and two eSense meters, which measure attention and meditation. Another feature, Poor Signal, provides signal quality information for users. Ideally, the value of Poor Signal should be zero. All the spectrum features are calculated from the raw EEG values by an algorithm inside the integrated circuit, updated with a frequency of one Hz. However, the sampling rate of the raw EEG values is 512 Hz.

Three PPG-type sensors were selected for HR monitoring from six modules designed by three companies. All three products have one LED to emit green light and one phototransistor to receive the reflection from the veins. Since this low-cost system could be applied in field studies in the future, the ease of applying the sensor on the human body and its esthetic appearance were considered. Therefore, chest strap products were excluded. Ear clip sensors were also excluded because of their unattractive appearance, which could discourage people from participating in research. Consequently, the Heart Rate Monitor Sensor for Arduino, Pulse Sensor, and Grove-Finger-Clip Heart Rate Sensor with a shell were selected for the next activity.

Moreover, only one GSR device designed by SEEED is available in the market. Grove-GSR Sensor measures the resistance of humans. However, the value measured by a sensor is noisy. Therefore, a high-pass filter should filter the data before being applied. This research used a simple moving-average method to reduce the effect of the white noise generated by the sensors (EEG and GSR). Although the unit of measured GSR value is intensity, the manufacturer offers a formula to convert an intensity value to Ohm.

Thus, this study selected one EEG, one GSR, and three HR sensors from the discrete physiological sensor for Arduino makers in the design and development activity.

#### 3.1.2. Activities 4 and 5: Demonstration and Evaluation

We utilized the library provided by the HR and GSR sensor manufacturer to develop a simple program in Arduino Studio to test the functionality. The EEG test program was developed using Python. In addition to the desired function, the quality and stability of the biometric signal were evaluated. Furthermore, the generated signal had to be clear and without interruption for two hours. User feedback on the comfort of wearing the sensor was also considered. Only when all of these criteria were satisfied, a specific physiological sensor was adopted.

The TGAM module was excluded from the current research because the value of Poor Signal was not zero most of the time during our trials. In addition, the values of the EEG signals oscillated significantly during connection. These signals were gathered by a program using NeuroSkyPy, a Python package for the NeuroSky TGAM module in the Python Package Index (PyPI), with Python 3.10, and executed in console mode.

A similar issue with EEG occurred with the HR sensors. Although all three products successfully measured people’s heart rates, they did not work constantly. Sometimes, people’s heart rate was lower than 50 beats per minute. Consequently, the three devices were unable to sense people’s heart rates at times. Since all three candidates were too unstable to work properly, the signal quality was poor. Therefore, the heart rate monitor for Arduino was not applied to the low-cost biofeedback system in the current study.

Fortunately, the GSR test result with members from our laboratory satisfied the desired criteria. Therefore, this GSR sensor for Arduino makers was chosen for the low-cost biofeedback system.

To summarize, GSR is the only sensor for Arduino makers used in this study. The study also considered EEG and heart rate monitor sensors from consumer-grade devices on the market. The development process iterated back to activity three.

### 3.2. Using Consumer-Grade Biofeedback Devices

#### 3.2.1. Activity 3: Design and Development

There are numerous consumer-grade EEG devices available on the market, including products from NeuroSky, Emotiv, Interaxon, and OpenBCI, as suggested by Sawangjai and colleagues [19]. The most critical condition for the current project was PyPI open-source library support. Table 2 summarizes the characteristics of the libraries for the target EEG in this study. For an open-source package, the essential consideration is whether a specific team maintains and documents the usage of the library. The version, release date, and documentation of the library on the website are crucial features to consider. The second condition was that the implemented library operation system had to be compatible with the platform used in this study, namely, Windows. All four products satisfied the requirements of the current study.

**Figure 2:**
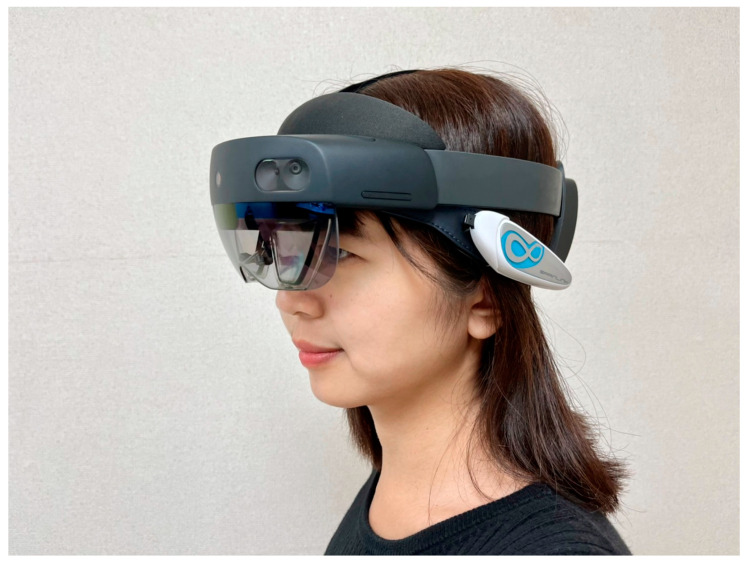
The participant wore HoloLens 2 and BrainLink lite.

The next step was to physically evaluate the hardware. All the target products are available on the local market. However, only NeuroSky has agents in Taiwan, making it the only product we could physically evaluate before purchase. BrainLink Lite is a headband-type EEG that uses NeuroSky’s TGAM, designed by Macrotellect. The electrodes in the headband make firm contact with the forehead, so the user does not feel pain like with other products that use ear clip electrodes for the ground or referred potential. The TGAM module integrates Bluetooth and chargeable lithium cells, and packages these in a tiny plastic shell, making the product light for users to wear. Consequently, users feel more comfortable than with other EEG products. BrainLink’s light, thin, and tiny features make it compatible with head-mounted MR headsets, such as HoloLens 2 (Figure 2). Thus, it is hoped that BrainLink Lite can be used to collect EEG data on the user experience evaluation of XR.

Heart rate monitors have become popular in recent years. Not every device displays heart rate information on the hardware display, but all the products share information through Bluetooth Low Energy (BLE) with the specific GATT specification. Developing a low-cost and open-source biometrics system could take advantage of BLE’s standard heart rate specification to simplify integration.

Although wristband-type products with heart rate monitor functions are prevalent in the market, most of them can access the information only from the software or mobile application provided by the manufacturers. Commerce competition limits the alternatives of heart rate application in low-cost and open-source biometrics systems. Nevertheless, the patented optical sensor technology in the Rhythm+2.0 heart rate monitor utilizes green and yellow LEDs to measure blood flow for a highly accurate reading with all skin tones [35]. A built-in accelerometer, which is applied to solve the motion artifacts issue caused by human movement [27], assists in providing hyper-accurate measurement. Since the yellow LED improves measurement accuracy for Asian people, the current study considered Rhythm+2.0 as the heart rate monitor for the low-cost, open-source biometrics system.

#### 3.2.2. Activity 4 and 5: Demonstration and Evaluation

A demonstration involved using the Python code developed earlier for the NeuroSky TGAM device to ensure stable signal quality index (i.e., Poor Signal = 0). This confirmed the suitability of BrainLink Lite for this study.

Additionally, a BLE communication program was developed in Python using the bleak library to validate the readings of the Rhythm+2.0 device, which showed heart rate values between 60–90 beats per minute for various members in our laboratory during the validation stage. As the readings were reasonable for individuals in a benign condition, the Rhythm+2.0 device was utilized in the current study.

All the required biofeedback devices were selected based on the nominal procedure of the DSRM paradigm, and the project iterated back to the final stage of the design and development activity.

### 3.3. Building a Low-Cost Biofeedback Platform

#### 3.3.1. Activity 3: Design and Development

Another biometric feature, emotion reflected on a person’s face, can be quickly captured using a webcam. Any webcam connected to a computer is generally capable of this function. However, this study employed a mainstream commercial product, Logitech StreamCam, to access users’ faces.

Once all the psychophysiological devices were determined, an integrated biofeedback system was developed using Python 3.10. The system featured a graphic user interface (GUI) for excellent usability. The GUI allows the user to input the required information, such as experiment name, treatment of experiment, participant identification, and experiment run. After the necessary data are entered, the sequential operations of the biofeedback system can be performed by clicking ordered buttons from left to right. As the sensors communicate with the integrated system, the psychophysiological signals are displayed on the GUI to make the users aware of the sensors’ working status under experimental conditions. All the signals were respectively represented on a trend chart, and the graphics were updated dynamically with a frequency of one Hz. However, the sampling frequency of the biometrical signal depended on the manufacturer’s design.

This study used BLE as the communication protocol of the low-cost system, in line with the survey results in Section 1.1. This is because BLE is recognized as an essential part of the application of wearable sensors. Apart from the physiological sensors mentioned earlier, an ESP32 development board, WEMOS LOLIN32, was used to broadcast the measured value of the GSR sensor. WEMOS LOLIN32 is equipped with features such as BLE communication, it is tiny in terms of size, and is rechargeable using 3.7-volt lithium cells. The GSR value was broadcast with a customized GATT using this package at a frequency of 125 Hz. Its small size was essential for a wearable device, while the rechargeable 3.7-volt lithium cells were light and easy to apply. The experimenter could quickly recharge the battery through the USB-D type interface.

**Figure 3:**
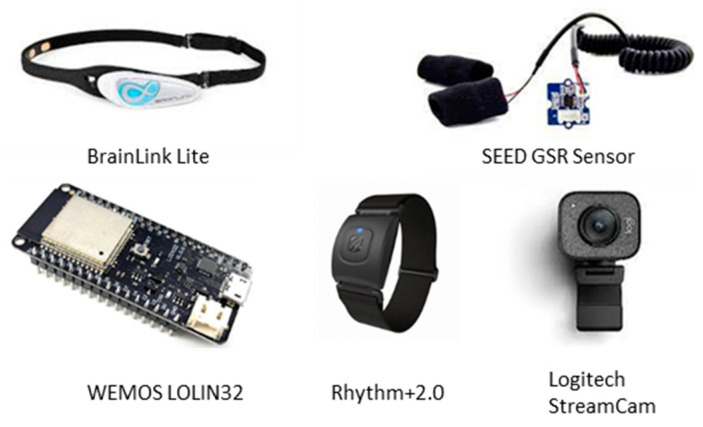
The biometrical sensor, devices, and development board used in this study. The total cost of these devices was about USD 800, which is a significant cost saving when compared with a medical-grade system.

Figure 3 shows the sensors/devices used in this research. Our solution costs less than USD one thousand compared with any medical-grade biofeedback system. Table 3 summarizes the free libraries that were used in the low-cost biofeedback system. The sampling frequency of each physiological characteristic is also listed.

**Figure 4:**
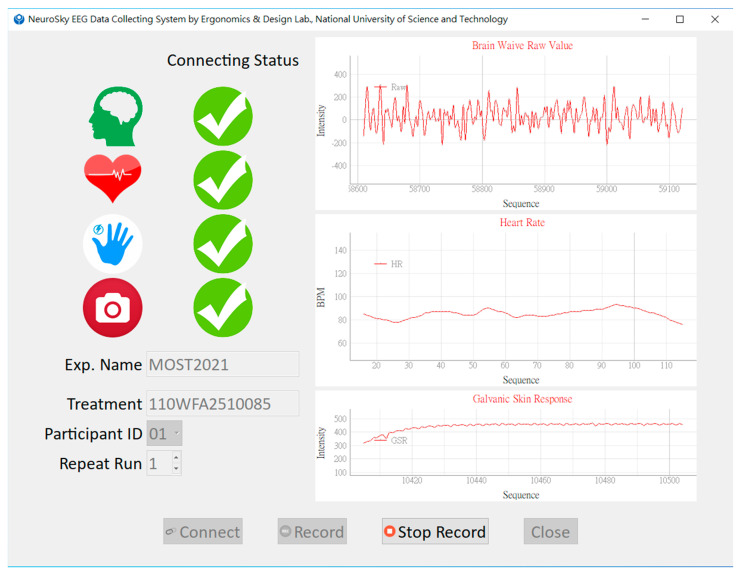
GUI of the low-cost biofeedback platform developed in this study.

To make the system easy to use, we designed a GUI (Figure 4) using PySide6 to interact with the experimenter. EEG data were collected through COM port using NeuroSkyPy; HR and GSR values were accessed via BLE GATT using bleak; and face images were captured via a webcam and processed using OpenCV-python. All the data were visualized through dynamic graphs utilizing pyqtgraph. The graphs on the GUI were updated every second, with each update renewing 512 readings of EEG raw value, 125 readings of GSR intensity, and one reading of HR (Figure 4). Table 4 shows the definition of the EEG powers derived by NeuroSky TGAM. Although the powers and eSense meters were not shown on the GUI, they were recorded and saved with other signals when the save button was clicked. Moreover, the software applied a multithreaded framework to integrate these signals effectively.

#### 3.3.2. Activity 4: Demonstration

To validate the low-cost, open-source biometric integrated system developed in this study, we conducted a simple experiment with one participant. The experiment consisted of one baseline lasting one minute and two tasks lasting ten minutes each. The tasks were designed to test the system’s ability to handle different circumstances.

**Figure 5:**
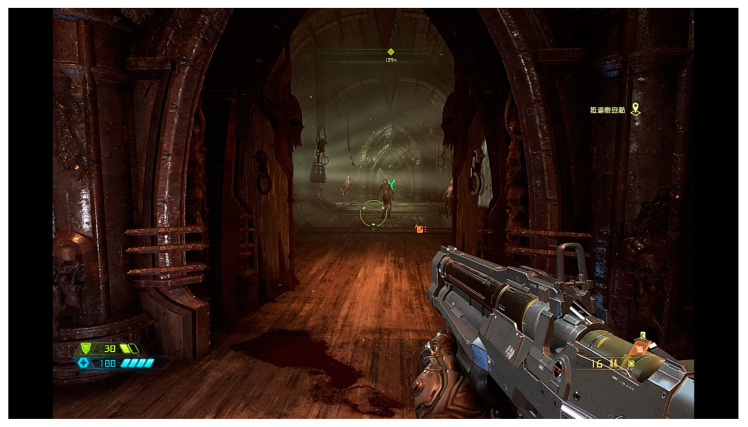
Screenshot of the first-person shooting 2D game used in this study.

The first task involved playing a popular first-person shooter (FPS) game, DOOM Eternal, using a physical keyboard and mouse. This task was referred to as the “2D game” since the screen is a two-dimensional environment. Figure 5 shows a screenshot of the game.

**Figure 6:**
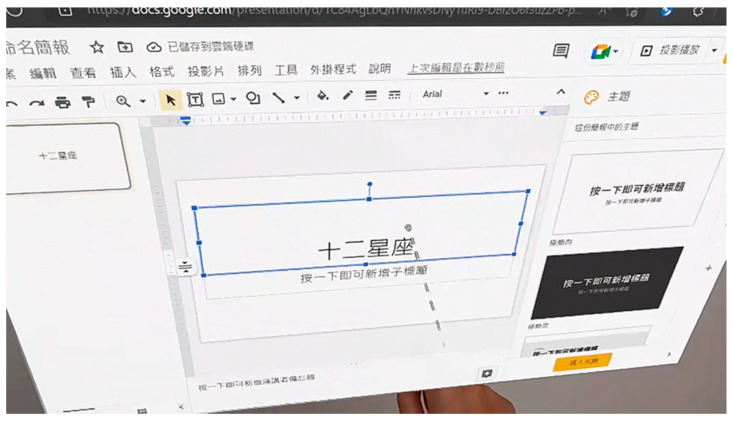
Screenshot of the 3D Google Slides captured through HoloLens 2.

The second task was to prepare the presentation slides using Google Slides in a mixed-reality (MR) environment. This task, which was called “3D slide” for MR science, involved a 3D environment. Figure 6 shows the screen when a user tries to prepare slides using HoloLens 2.

There are no reports in the literature regarding the emotional response to using HoloLens 2 to create a slide. However, all the members who participated in the trial run complained about the task and provided feedback that was very different compared with the 2D game. We were only able to confirm that the 3D slide elicits a different response to the 2D game.

Previous research [88] has shown that participants with varied experience levels perceived a more positive than negative affect in three FPS games, including the previous version of DOOM. As a result, the shooting game was assumed to create a positive emotional user experience.

### 3.4. Activity 5: Evaluation

**Figure 7:**
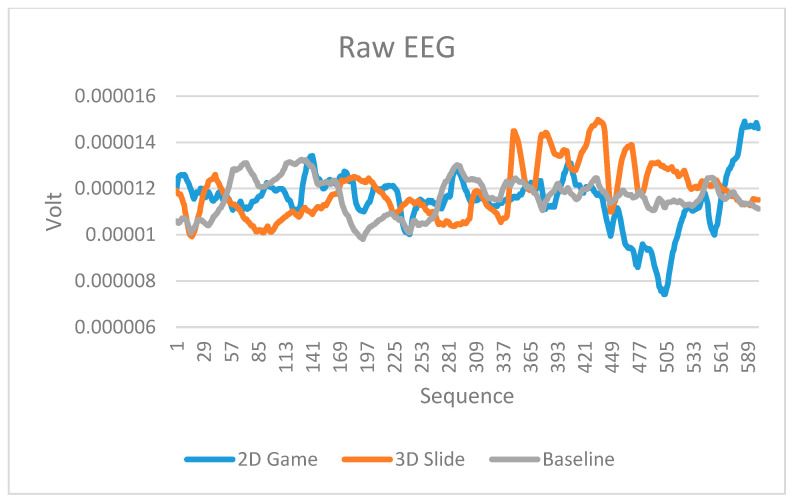
Raw EEG readings in the baseline, 2D game, and 3D slide.

**Figure 8:**
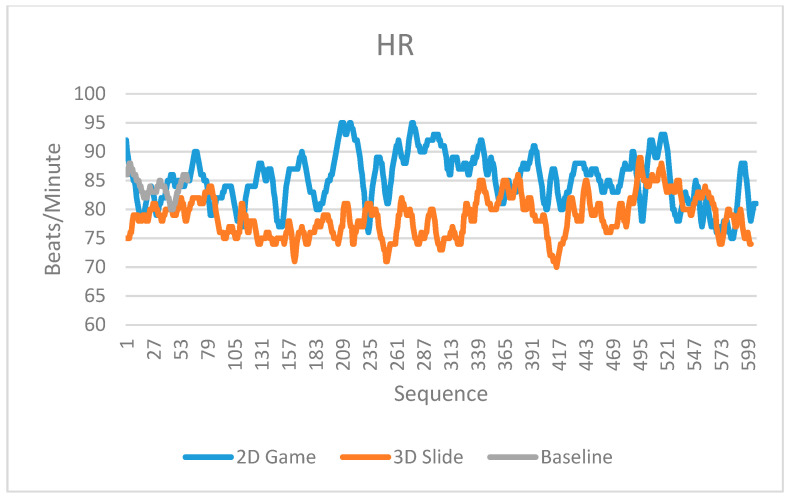
Heart rate readings in the baseline, 2D game, and 3D slide.

**Figure 9:**
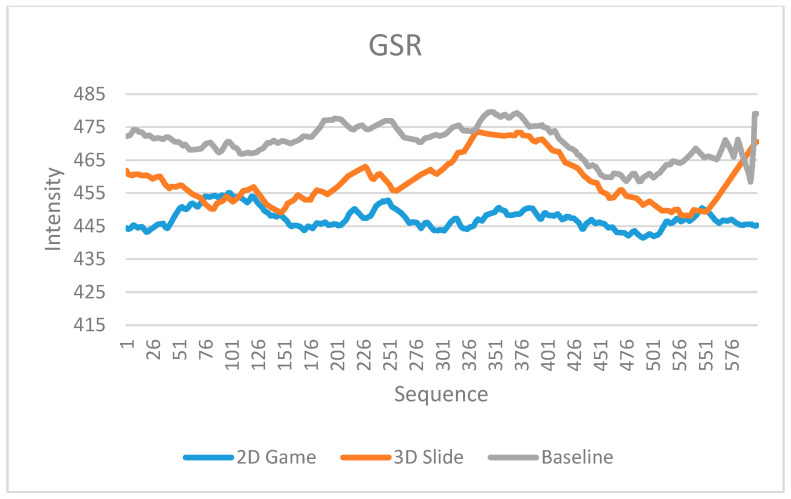
GSR readings in the baseline, 2D game, and 3D slide.

All of the physiological signals collected by the low-cost biofeedback system showed differences between the two designed scenarios in the experiment. Figure 7, Figure 8 and Figure 9 display the EEG raw values, HR, and GSR, respectively. Each signal has readings in the baseline, the 2D game, and the 3D slide, respectively. The unweighted mean number of data points is 100 for EEG and 125 for GSR. Because there is a considerable amount of data in the EEG raw values and GSR, it means the differences are not easy to observe in the limited width of a graph. Therefore, only the first 600 data points (aligned with the number of HRs) were truncated and displayed in Figure 7 and Figure 9.

In addition, this study conducted two sample t-tests based on the data in Figure 7, Figure 8 and Figure 9. Each signal had three types of t-tests (baseline vs. the 2D game, baseline vs. the 3D slide, and the 2D game vs. the 3D slide). Table 5 summarizes the nine t-test results. All significant differences (*p*-value < 0.05) indicate that the biofeedback system developed in this study has the potential to be applied in future studies.

**Figure 10:**
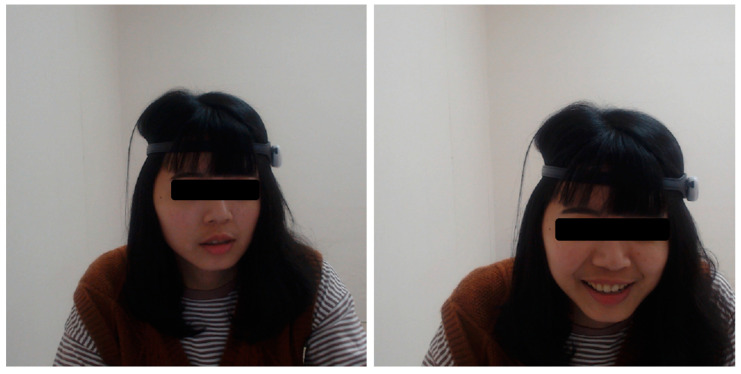
Facial expression of the participant was neutral at the beginning of the 2D game (**left**) and smiling during the 2D game (**right**).

Although the derived EEG power signals are not shown in Figure 6, Table 6 displays the saved powers. The derived power signals were updated with a frequency of one Hz and had no noise (Poor Signal is zero). Figure 10 shows the users’ facial expressions at the beginning of the 2D game and during the 2D game, respectively. A smiling face during the game experience is distinguished from a neutral face before the game starts.

## 4. Discussion

### 4.1. Considerations of Selecting Sensors for Arduino Makers

When selecting EEG products, researchers should take into account the utility frequency. There are two power line frequencies used worldwide: 50 Hz and 60 Hz, depending on the region or country. If an EEG device uses a different frequency from the electricity system in the researcher’s region or country, the signal will be biased from the correct value due to noise. The TGAM module for Arduino makers in this study is designed for users with a 50 Hz utility frequency. Therefore, the poor signal was not zero, indicating the presence of noise because the power frequency in Taiwan is 60 Hz.

There are many PPG-type HR sensor options available in the makers’ market, and every manufacturer provides example codes and libraries for their users. However, the stability of acquiring the signal successfully and the accuracy of the reading value are not reported by the manufacturers. Due to the limitations of PPG technology, researchers have suggested using an LED matrix instead of a single LED to increase accuracy and stability [27]. Therefore, manufacturers targeting Arduino makers should endeavor to improve reliable measurement with more LEDs. However, consumer HR monitors with at least two LEDs are popular. The Rhythm+2.0 used in this study uses an extra yellow LED to obtain a more accurate measurement for Asian people [35].

All consumer HR monitors support the BLE function with a unique UUID, which makes accessing data with mobile equipment through applications quick. Unfortunately, most mainstream products can only be accessed through the specific software provided by the manufacturer. As a result, researchers wishing to integrate consumer-grade HR devices into their research face data processing difficulties. Investigators are usually interested in specific events in their research, and precise logging of the start/end time point when the onset occurred is complex, requiring more effort when using this type of product in research.

### 4.2. Performance of the Low-Cost Biofeedback Platform Developed in This Study

While no noise obstructs the EEG signal with a 60 Hz chip, the poor signal is always zero (Table 6). The waveform obtained from the BrainLink Lite device (Figure 4) closely resembles those reported in the literature. The raw values obtained from the two different tasks show differences (Figure 5), indicating that the single-electrode wireless EEG can be applied in human behavior studies. This finding is consistent with the literature [89,90]. Although it is difficult to explain the meaning of EEG raw values directly, a noticeable difference exists (Figure 7). Further power analysis (Table 6) could help to explain the results more comprehensively.

In contrast, the heart rate (HR) readings can directly explain the relationship between experimental settings. For example, the heart rate of the 2D game (Figure 8) was consistently higher than for the 3D slide, which suggests that the 2D game elicits more excitement from the player.

Similarly, the GSR sensor for Arduino makers clearly distinguished between the 2D game and the 3D slide. Unlike the HR, the GSR signal in the 2D game was consistently lower in intensity than the signal in the 3D slide (Figure 9). This finding suggests that the user may have felt more relaxed during the 2D game than during the 3D slide. This is because the former is more enjoyable, while the latter is more frustrating. Our results demonstrate that HR and GSR can be used to evaluate whether the user experience is consistent with the literature [49].

In addition, facial expressions captured by a regular webcam and a mixed-reality headset have been validated as a means of emotion recognition in UX studies [52]. Figure 10 shows the facial expressions in neutral and delighted states during the 2D game in our study. Furthermore, the results of the EEG, HR, GSR, and facial expressions validate that the signals captured by our low-cost biofeedback system match the designed experimental conditions. Therefore, the platform developed in this study has the potential to be applied in future UX studies, which satisfies the first aim of our study.

While low-cost biofeedback studies have been reported in the literature, their number is limited. Moreover, the existing literature reports that the hardware used was limited [1]; had a simple user interface for recording, event-marking, and downloading data without integration with the experiment control [91]; or required another computer to execute the experiment [26]. Some studies, such as event-related potential research, require precise biometric accuracy in milliseconds at the occurrence of the event of interest [92]. However, the existing literature cannot meet this specific requirement.

This study aimed to integrate low-cost biofeedback hardware with graphic user interface software using open-source libraries to demonstrate its ability to differentiate between design contexts. Biofeedback was used as standalone equipment, without integration with the experiment stimulus. However, the GUI was an object-oriented programming class that can serve as an SDK. Researchers can customize the SDK with their experiment stimulus and synchronize it if necessary, allowing for precise biometric recordings when the desired event occurs in the future. This study achieved its second aim by integrating data collection and experiment control within one single computer system.

While consumer-grade wearable devices have been widely used in non-medical applications, researchers need to understand whether the devices they choose satisfy the technical requirements of their study objectives. Compared with medical-grade instruments, consumer-grade products have lower sampling rates, less accuracy, a time lag in real-time data streaming, and may even experience data loss, as indicated in the introduction of this paper. These limitations can result in research failing to achieve desired outcomes.

Researchers can utilize the wearable and lightweight biofeedback system to conduct laboratory or field research. Moreover, the low-cost platform’s convenience facilitates the construction of a database based on physiological signals for the development of machine learning models. This database is crucial for affective computing that is used in various fields, such as ergonomics, human factors engineering, user experience, human behavioral studies, and human–robot interaction.

## 5. Conclusions

Although self-developed physiological data collection systems are reported in the literature, they typically consist of either a single sensor for collecting one type of signal or multiple sensors that are used independently with various personal computers and software to collect data To the best of our knowledge, the present study is the first to emphasize the importance of integrating low-cost individual sensors and consumer products into a single biofeedback system, developed using open-source software, to create a reusable SDK for future studies.

This low-cost biofeedback solution is still at an experimental stage, and more research is required to investigate integrated experimental stimuli. Additionally, other physiological signals, such as electromyography (EMG) or eye-tracker, should be considered in future efforts to integrate the biofeedback platform. Wireless EMG sensors are readily available in the Arduino makers’ market, while all mainstream eye-tracker manufacturers have recently supported open-source Python libraries. A PyPI eye-tracking library for general webcams is also available. The open-source movement has enabled the availability of a diverse and rich low-cost biofeedback platform.

A summary of the significant contributions of the current study are listed below.

1. Our results demonstrate the possibility of identifying different physiological signals in varied circumstances. This result suggests the potential for using a low-cost biofeedback system in non-medical research, such as ergonomics, human factors engineering, user experience, human behavioral studies, and human–robot interaction.
2. Using the self-developed system, researchers can integrate the biofeedback platform with different stimuli in various research contexts. By simultaneously recording the time points of various events and physiological signals, researchers can reduce the effort required for post-data processing while increasing the accuracy of time alignment to a specific event.
3. Instead of using expensive medical-grade products, the success of the low-cost biofeedback system can serve as a reference framework, benefiting researchers who have limited budgets for equipment and biofeedback system development.
4. The lightweight hardware makes the devices convenient to wear in ambient laboratories or in field studies.

## Figures and Tables

**Table 1:** Available EEG, HR, and GSR sensors for Arduino makers in the local market.

Sensor	Manufacturer	Model
EEG	Twarm.com	NeuroSky TGAM MDL0026
GSR	SEEED	Grove-GSR_Sensor V1.2
HR	SEEED	Grove-Finger-clip Heart Rate Sensor
	SEEED	Grove-Ear-clip Heart Rate Sensor
	SEEED	Grove-Chest Strap Heart Rate Sensor
	DFRobot	Heart Rate Monitor Sensor (PPG)
	DFRobot	Analog Heart Rate Monitor Sensor (ECG)
	World Famous Electronics LLC.	Pulse Sensor

**Table 2:** Available EEG library for targeted consumer devices in PyPI.

Designer	Library	Version	OS	Release Date	DOC
NeuroSky	NeuroSkyPy	1.6	Windows	24 January 2020	Yes
Emotiv	pyeeg ^1^	0.0.2	Windows	31 March 2021	No
Interaxon	Muselsl	2.2.1	Windows,	2 June 2022	Yes
			Mac,		
			Linux,		
			POSIX,		
OpenBCI	pyOpenBCI	0.13	Windows	28 May 2019	Yes
			Mac,		
			Linux ^2^		

^1^ Only supports 5 channel products. ^2^ Ganglion only runs on Linux. Cyton runs on all OSs.

**Table 3:** Libraries used for the biofeedback system developed in this study.

Function	Library	Version	Sampling/Updating Frequency
GUI	PySide6	6.3.0	1 Hz
EEG	NeuroSkyPy	1.6	512 Hz for the raw value and 1 Hz for EEG power
HR	bleak	0.14.2	1 Hz
GSR	bleak	0.14.2	125 Hz
Face	OpenCV	4.5.5.64	1 Hz
Graph	pyqtgraph	0.12.4	1 Hz

**Table 4:** EEG powers derived from NeuroSky TGAM.

Power	Range
delta	0.5–2.75 Hz
theta	3.5–6.75 Hz
low-alpha	7.5–9.25 Hz
high-alpha	10.0–11.75 Hz
low-beta	13.0–16.75 Hz
high-beta	18.0–29.75 Hz
low-gamma	31.0–39.75 Hz
mid-gamma	41.0–49.75 Hz

**Table 5:** Two sample t-test for HR, GSR, and EEG raw data use paired combinations for the baseline, 2D game, and 3D slide conditions.

Comparisons	*t-*value	*p*-value
HR: 2D game vs. baseline	t_658_ = 2.24	0.026
HR: 3D slide vs. baseline	t_658_ = −11.17	<0.001
HR: 2D game vs. 3D slide	t_1198_ = 27.85	<0.001
GSR: 2D game vs. baseline	t_1198_ = −91.47	<0.001
GSR: 3D slide vs. baseline	t_1198_ = −31.74	<0.001
GSR: 2D game vs. 3D slide	t_1198_ = −36.70	<0.001
EEG Raw: 2D game vs. baseline	t_1198_ = −2.02	0.043
EEG Raw: 3D slide vs. baseline	t_1198_ = 4.17	<0.001
EEG Raw: 2D game vs. 3D slide	t_1198_ = −5.28	<0.001

**Table 6:** Derived EEG powers dataset (part).

Sequence	Poor Signal	Attention	Meditation	Delta	Theta	High Alpha	Low Alpha	High Beta	Low Beta	Mid Gamma	Low Gamma
0	0	66	77	2414	4479	1085	3016	2274	7953	3891	4919
1	0	48	81	225175	207487	39649	27987	24423	15753	49699	11359
2	0	50	67	89096	26396	4230	7523	9308	4242	3581	5368
3	0	40	51	485739	659212	59886	134022	52701	71614	71203	31691
4	0	40	56	86695	45658	15004	30145	25936	24866	31767	25100
5	0	57	47	11212	29726	4074	2742	35128	11414	13355	37743
6	0	69	38	18924	18845	4427	5056	39467	27661	14828	21849
7	0	84	35	51547	48415	12819	9886	30886	37115	19827	21989
8	0	78	29	304630	113782	2388	30708	13421	11459	8432	22863
9	0	61	34	706916	266871	11765	32688	26741	9476	8145	21529
10	0	57	37	368630	24306	7170	5785	39560	10214	53417	37668
11	0	61	38	6043	20863	8923	7053	26674	20595	31412	23924
12	0	74	34	121654	20911	3792	2298	13380	5358	7991	4227
13	0	83	34	793945	107148	7736	13083	43273	12841	25614	52645

## Data Availability

Not applicable.
